# Does hip protector prevent falls and hip fractures? An umbrella review of meta-analyses

**DOI:** 10.1186/s12877-024-05122-x

**Published:** 2024-06-12

**Authors:** Qingchen Da, Yingheng Xiao, Feng Wu, Yueliang Chen, Liping Li

**Affiliations:** 1 School of Public Health, Shantou University, Shantou, 515041 China; 2 Injury Prevention Research Center, Shantou University Medical College, Shantou, 515041 China

**Keywords:** Hip fractures, Hip protectors, Falls, Older adults, Umbrella review

## Abstract

**Background:**

Wearing hip protectors is a measure used to prevent hip fractures caused by falls. However, its protective effect has remained controversial in previous studies. This study provides a rationale for the use of hip protectors by pooling all the current meta-analysis evidence.

**Methods:**

We conducted an umbrella review of all the current meta-analysis articles about the efficacy of hip protectors to reduce hip fractures and falls in communities and/or institutions. Major databases including EMBASE, Cochrane Library, PubMed and Web of Science, were searched up to June 2022. Two reviewers screened the studies, extracted the data, and conducted the methodological quality assessment independently. The primary outcome was the association statistic (odds ratio (OR), relative risk (RR), etc.) reported in the meta-analysis that quantified the influence of the intervention on hip fractures and falls compared to that of the control group. Narrative synthesis was also conducted. Forest plots and the AMSTAR score were used to describe the results and quality of the pooled literature, respectively.

**Results:**

A total of six meta-analysis articles were included in the study. Hip protectors were effective at reducing hip fractures in older individuals who were in institutions (nursing or residential care settings) but not in communities (RR = 0.70, 95% *CI* 0.58 to 0.85, *I*^*2*^ = 42%, *P* < 0.001) (RR = 1.12, 95% *CI* 0.94 to 1.34, *I*^*2*^ = 0%, *P* = 0.20), and they did not reduce falls (RR = 1.01, 95% *CI* 0.90 to 1.13, *I*^*2*^ = 0%, *P =* 0.89).

**Conclusions:**

Hip protectors are effective at preventing hip fractures in institutionalized older adults but not in community-dwelling older adults.

**Trial registration:**

This study has been registered in PROSPERO (PROSPERO ID: CRD42022351773).

**Supplementary Information:**

The online version contains supplementary material available at 10.1186/s12877-024-05122-x.

## Introduction

Falls is a common public health problem and a common cause of disability and death in the older individuals worldwide [1, 2]. Falls poses such a heavy health burden because it can lead to hip fractures, which is an extremely troublesome clinical problem. Studies reveal that more than 90% of hip fractures are caused by falls [3]. Hip fractures are the most serious type of fracture [4] and are the most common cause of hospital admission due to the use of acute orthopedic wards in older adults [5]. As the number of older people increases, the number of hip fractures is increasing [6], and the number is expected to increase from 1.66 million to 6.26 million worldwide from 1990 to 2050 [7]. Hip fractures can have adverse effects on patients. The highest mortality rate within 6 months is associated with hip fracture [8] and the mortality rate within one year is approximately 17–33% [6]. The main causes of death are “septicemia, pneumonia/influenza and digestive system disorders” [9]. Hip fractures also result in decreased independence, disability, chronic pain, fear of falling, and difficulty walking [10–12]. Therefore, preventing hip fractures caused by falls is urgently needed.

The mechanism of hip fracture is the impact force (5600 N) generated during a fall that exceeds the fracture threshold (2100 N) [4]. The human body itself can provide some protection through the soft tissues of the hip by absorbing the energy generated by the fall and reducing the impact force [13, 14]. However, the protective effect decreases with age as the soft tissues of the hip increase in stiffness, among which is the greater trochanter of the femur the most [15]. Since most hip fractures are caused by lateral falls and affect the greater trochanter [16], the incidence of these fractures can be reduced by reducing the impact of lateral falls [12]. The hip protector is a device that protects the hip by reducing the force of a lateral fall to below the fracture threshold [6, 17]. Primarily used to prevent and reduce the incidence of hip fractures, hip protectors are usually pairs of hard or soft pads that cover the greater trochanter area and fit in the pocket of specially designed underpants [10, 12, 18]. The hard pad uses a hard pad that shunts the force of impact from the greater trochanter to the soft tissues around the femur, while the soft pad uses compressible material that absorbs energy [19–21]. Previous studies have shown its efficacy, in which it has been reported that wearing hip protectors reduces the risk of hip fracture by nearly three times compared to not wearing hip protectors [22].

However, previous meta-analyses have shown that the effectiveness of hip protectors for fall prevention is controversial [23, 24]. Therefore, there is a need for a comprehensive review of all relevant meta-analysis papers, i.e., an umbrella review. An umbrella review is a summary of the evidence on several relevant clinical issues from multiple meta-analyses, providing support and assistance for decision making [25].

The aim of this umbrella review is to provide a systematic overview and appraisal of meta-analyses investigating hip protectors against falls and hip fractures in (a) community-dwelling and (b) institutional (including nursing or care homes) older adults.

## Methods

### Eligibility criteria

Types of study to be included: meta-analyses of hip protector intervention to reduce hip fractures and falls.

Types of outcome measures: The primary outcome was the association statistic (odds ratio (OR), relative risk (RR), etc.) reported in the meta-analysis that quantified the influence of the intervention on hip fractures and falls compared to that of the control group.

### Search method

Two authors (Q.D. and Y.X.) searched the following electronic databases: EMBASE, Cochrane Library, PubMed and Web of Science from inception till June 2022. A third author (F.W.) was available as a mediator.

The key words used in the searches were ‘hip protector’ AND ‘meta-analysis’.

We considered the reference lists of all potentially eligible article (Supplementary Material 1). We considered only meta-analyses that were derived from a systematic review of the literature without any restriction in languages. When meta-analyses reported multiple subgroups and sensitivity analyses, we reported the main effect sizes of the interventions. When encountered a meta-analysis that was an update of a previous review, we included only the most recent one. If we encountered reviews on similar topics, but containing different search strategies, inclusion criteria, analyses, and results, we included both reviews (at the discretion of the three authors). When encountered meta-analyses that included some randomized controlled trials accounted for ≥ 50% of the included studies, we included pooled results. We only incorporated the pooled analyses of individually and adjusted cluster randomized trials, when we encountered pooled analyses with both individually randomized controls and whole cluster randomized controls.

### Data extraction and analysis

Two reviewers (Q.D. and Y.X.) independently reviewed the titles and abstracts of potentially relevant papers identified through the search strategy. The full texts of all potentially eligible papers were reviewed before making a final decision on eligibility. A third reviewer (F.W.) was available for mediation.

All the data were extracted by two reviewers (Q.D. and Y.X.). The following data were extracted: first author, country, setting, population, aims of the study, inclusion criteria, fall and hip fracture rates, number of studies and participants included in the meta-analysis, association statistics of the intervention, heterogeneity, test for overall effect, adverse events, publication bias and authors’ conclusions.

A third reviewer (F.W.) was available for mediation for any disagreements in the data extraction.

When a meta-analysis included multiple outcome indicators, each outcome indicator was extracted separately for analysis.

### Statistical analysis

#### Assessment of heterogeneity

We estimated the summary effect size and its 95% confidence interval (95% CI) by the random-effects model. Heterogeneity in the meta-analysis was estimated with the I2 metric, with values between 50% and 75% indicating high heterogeneity and ≥ 75% indicating very high heterogeneity [26].

#### Data synthesis

We conducted separate umbrella reviews comparing interventions to reduce falls and hip fractures in community and institutional settings. Meta-analyses that met the inclusion criteria formed the unit of analysis. Only data available from reviews are presented. The results from the reviews were synthesized via a narrative synthesis, with tabular presentations of the findings and forest plots for reviews that performed a meta-analysis. Logarithmic transformation of the RR values and 95% CIs was performed to plot the forest plots. Summary tables describing the review characteristics and findings are also presented. All the statistical analyses were performed using R 4.2.1.

### Methodological quality assessment

Two authors (Q.D. and Y.X.) conducted the methodological quality assessment of all included meta-analyses with the AMSTAR, which is an 11-item methodological quality assessment tool [27]. Based on the AMSTAR score, the quality of the studies could be classified as high (8–11), medium (4–7) or low (0–3) [28, 29]. A third reviewer (F.W.) was available for mediation.

## Results

### Literature search

**Fig. 1:**
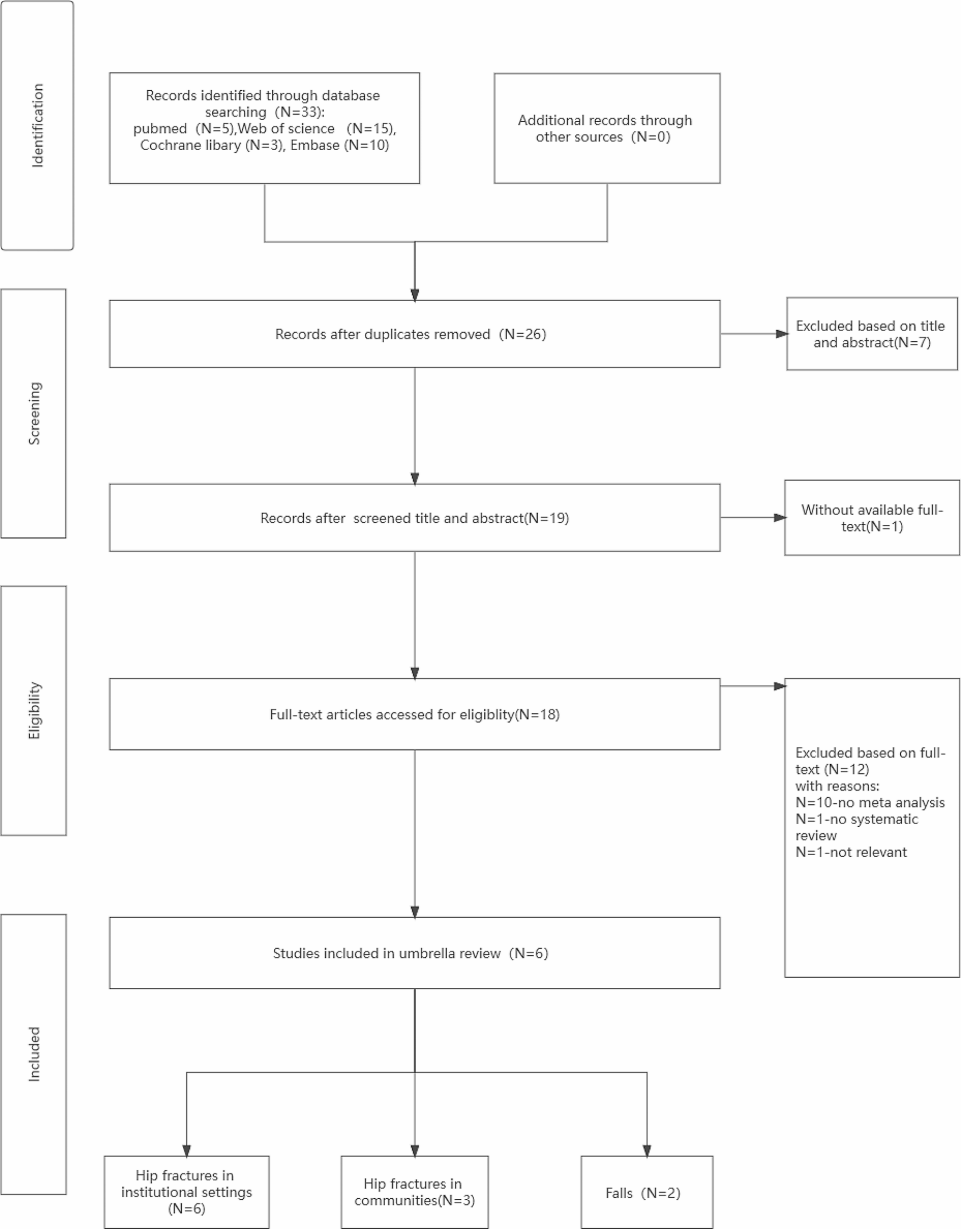
Flowchart of the literature search

A total of 33 articles were retrieved, and 27 articles were excluded according to the inclusion and exclusion criteria (Supplementary Material 1). Among the final samples, 6 unique meta-analyses were included. The full details of the search results are shown in Fig. 1.

### Methodological quality

Overall, the methodological quality of the included meta-analyses was moderate to high. Specifically, five studies were rated as high quality [5, 10, 12, 30, 31], and 1 was rated as moderate quality [32](Table 1). One meta-analysis did not formally assess heterogeneity by statistical tests [31], and the details of these heterogeneities are summarized in Table 2.

**Table 1:** Literature quality evaluation table

Study	Item 1	Item 2	Item 3	Item 4	Item 5	Item 6	Item 7	Item 8	Item 9	Item 10	Item 11
Waldegger, L,et al. 2003	Y	Y	Y	N	N	Y	Y	Y	Y	N	N
Sawka, A. M, et al. 2005	Y	Y	Y	Y	N	Y	Y	Y	Y	N	Y
Parker, M. J, et al. 2006	Y	Y	Y	Y	N	N	Y	Y	Y	N	Y
Sawka, A. M, et al. 2007	Y	Y	Y	N	N	Y	Y	Y	Y	N	Y
Oliver, D,et al. 2007	Y	Y	Y	Y	N	N	Y	Y	Y	N	N
Santesso, N,et al. 2014	Y	Y	Y	N	Y	Y	Y	Y	Y	Y	Y

### Summary of the article analysis results

The full details of the included meta-analyses are listed in Tables 2 and 3. In brief, six meta-analyses provided data on hip protector interventions in community and institutional settings [5, 10, 12, 30–32]. Three of the studies included evidence in both institutional and community settings [5, 10, 12] ; others included evidence only in institutional settings such as care homes and nursing homes [30–32]. The meta-analysis included 3 [32] to 16 [12] studies involving 1480 [32] to 11,808 [12] different participants. Only 2 meta-analyses have examined the effect of hip protectors on fall prevention [12, 30].

**Table 2:** Summary findings for each effect size for the hip protectors

Author	Year	Studies	Participants	Outcome	Setting	Effect size	Effect size	Effect size (95% CI)	Heterogeneity	Test for overall effect
Waldegger, L,et al.	2003	3	1480	hip fracture	nursing home	RR	0.40	0.23–0.70	*P* = 0.94	*P* = 0.001
Sawka, A. M, et al.	2005	4	5696	hip fracture	communities	RR	1.07	0.81–1.42	chi-square = 2.13, *P* = 0.55	*P* = 0.62
		3	1188	hip fracture	institutionalized	RR	0.56	0.31–1.01	NA	NA
Parker, M. J, et al.	2006	11	9859	hip fracture	institutional setting (nursing or residential care settings)	RR	0.77	0.62–0.97	chi-square = 16.64, df = 10, *P* = 0.08, *I*^*2*^ = 39.9%	*P* = 0.03
		3	5135	hip fracture	communities	RR	1.16	0.85–1.59	chi-square = 1.92, df = 2, *P* = 0.38, *I*^*2*^ = 0%	*P* = 0.36
Sawka, A. M, et al.	2007	4	1992	hip fracture	nursing	OR	0.40	0.25–0.61	NA	NA
Oliver, D ,et al.	2007	11	NA	hip fracture	care home	RR	0.67	0.46–0.98	*I*^*2*^ = 39%	NA
			NA	falls	care home	RR	0.97	0.77–1.22	*I*^*2*^ = 90%	NA
Santesso, N,et al.	2014	14	11,808	hip fracture	institutional setting (nursing or residential care settings)	RR	0.82	0.67-1.00	chi-square = 19.29, *P* = 0.11, *I*^*2*^ = 32.62%	*P* = 0.05
		4	5306	hip fracture	communities	RR	1.15	0.84–1.58	chi-square = 2.18, *P* = 0.7, *I*^*2*^ = 0%	*P* = 0.39
		16	11,275	falls	None	RR	1.02	0.9–1.16	chi-square = 198.69, *P* < 0.000, *I*^*2*^ = 92.45%	*P* = 0.74

**Table 3:** Summary findings for adverse events, publication bias and conclusions for hip protectors

Author	Aderver events	Publication bias	Conclusion
Waldegger, L,et al.	skin irritation	NA	Support the use of hip protectors in an institutional setting
Sawka, A. M, et al.	NA	NA	Not support the use of hip protectors outside the nursing home setting.
Parker, M. J, et al.	NA	NA	(1) Oppose the use of hip protectors at home (2) and not support the use of hip protectors in an institutional setting.
Sawka, A. M, et al.	NA	NA	Support the use of hip protectors in nursing homes.
Oliver, D ,et al.	NA	NA	Support the use of hip protectors in nursing homes.
Santesso, N,et al.	skin irritation	NA	Support the use of hip protectors in nursing homes.

### The efficiency of hip protectors for hip fracture

**Fig. 2:**
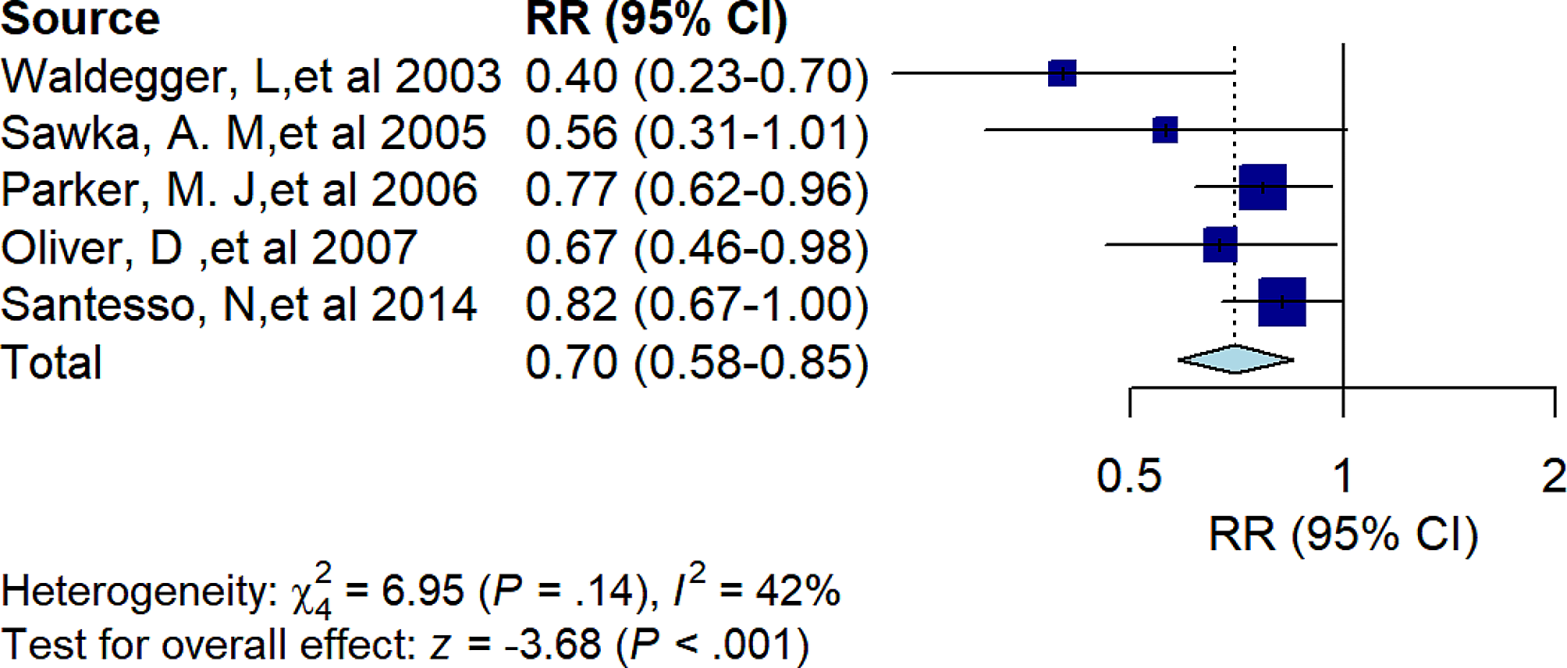
Forest plot showing the results of umbrella reviews of meta-analyses investigating the association between hip protectors and the risk of hip fractures in institutional settings (nursing or residential care settings)

Five studies have shown that hip protectors are effective at reducing the incidence of hip fracture in institutionalized older adults (RR = 0.70, 95% *CI* 0.58 to 0.85, *I*^*2*^ = 42%, *p* < 0.001) (Fig. 2). Another Bayesian meta-analysis showed that hip protectors reduced the incidence of hip fractures in nursing homes (RR = 0.40, 95% *CI* 0.25 to 0.61).

**Fig. 3:**
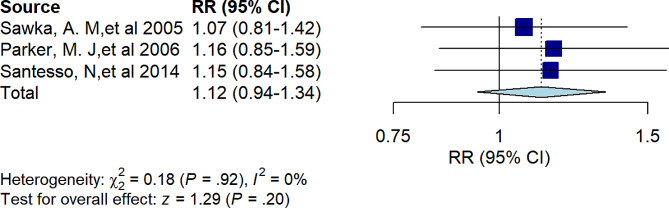
Forest plot showing the results of umbrella reviews of meta-analyses investigating the association between hip protectors and the risk of hip fractures in communities

Three studies found that hip protectors were not effective at reducing the incidence of hip fractures in community-dwelling older adults (RR = 1.12, 95% *CI* 0.94 to 1.34, *I*^*2*^ = 0%, *P =* 0.20) (Fig. 3).

**Fig. 4:**
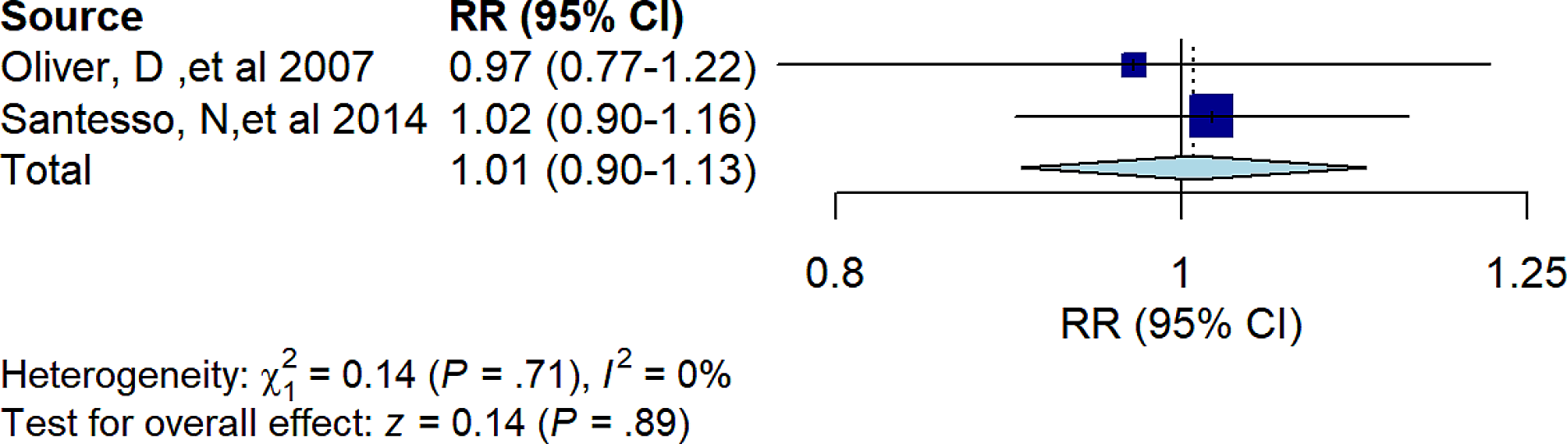
Forest plot showing the results of umbrella reviews of meta-analyses investigating the association between hip protectors and the risk of falls

Two studies found that hip protectors were not effective at reducing the incidence of falls (RR = 1.01, 95% *CI* 0.90 to 1.13, *I*^*2*^ = 0%, *P =* 0.89) (Fig. 4).

## Discussion

### Main results

Wearing hip protectors is an effective strategy for preventing and reducing hip fractures from falls in people at high risk. The use of hip protectors to prevent hip fractures was first studied in 1988, in which the number of hip fractures in subjects wearing hip protectors and those not wearing hip protectors were 0 and 4, respectively [33]. In 1993, the results of the first large randomized controlled trial worldwide showed that hip protectors reduced the risk of hip fracture (RR = 0.44) [13]. However, in recent years, as studies have been updated, conflicting results have emerged regarding the value of these devices for application; i.e., the use of hip protectors does not determine whether fracture reduction or improvement in quality of life can be achieved [23, 24]. To obtain evidence of better quality, this paper summarizes the available meta-analyses using an umbrella review.

The results showed that the use of hip protectors did not reduce the incidence of falls or hip fractures in community-dwelling older adults but did reduce the incidence in institutionalized older adults. This finding is consistent with previous studies [12]. On the one hand, it may be that institutionalized older adults are supervised by staff when using hip protectors; on the other hand, institutionalized older adults themselves are at high risk for hip fracture and are better protected by hip protectors [10]. The lack of protection in the community may be due to low adherence. The community participants cannot be supervised by staff to ensure adherence in the same way as in the institution, leading to relatively lower adherence. Research on measuring adherence in the community is lacking. Future studies may take into account measuring community adherence.

Poor adherence is a significant barrier to hip protectors use [34]. Studies have shown that the average adherence to hip protectors is less than 50% [35]. There are several factors that influence adherence. Studies have shown that common influencing factors include “not being comfortable (too tight/poor fit), the extra effort (and time) needed to wear the device, urinary incontinence and physical difficulties/illnesses” [11]. In particular, instead of avoiding falls and resulting hip fractures while wearing the hip protector, falls and fractures may occur during the process of putting on and taking off the hip protector due to difficulty in balance [36]. Therefore, the right use of hip protectors is of great importance. Besides, people who have difficulty using hip protectors need help from others to prevent injuries caused by using them on their own. The appearance of the hip protector itself is also a factor that affects adherence. The appearance of the hip protector may not be attractive to the user, leading to a refusal [11]. Also, the cost of hip protectors is a pivotal factor in adherence. Studies have shown that adherence can be improved if a free hip protector is available [37]. In addition, individuals with a history of falls may have a higher adherence rate [38]. On the one hand, this high-risk group may be more willing to use hip protectors themselves. On the other hand, caregivers may be more likely to urge individuals at risk of wearing hip protectors [13].However, research has demonstrated that adherence of wearing hip protectors decreased over time.After one month, adherence was recorded at 60.8%, whereas after 12 months, it approaches half of this initial rate. Furthermore, it was observed that most individuals did not wear their hip protectors during the night [39]. Therefore, it is imperative that we should improve adherence in the future.

The results of this study suggest that wearing hip protectors is suitable for preventing hip fractures in the institutional population. According to the recommendations of the International Hip Protector Research Group, the appropriate population for hip protectors is individuals at high risk for hip fracture, for which the annual incidence is > 3% [20]. Future research is needed to further define the appropriate population for hip protectors.

### Strength

First, compared to the previous umbrella review [40], we included evidence from older adults in the community and created forest plots to summarize the results across studies. Second, compared to the previous umbrella review [40], this study included more studies with larger sample sizes and was more convincing.

### Limitations

First, the included studies used different summary measures (e.g., OR and RR) to assess the effects of the intervention, which may have had some impact on the findings. In the future, researchers should consider harmonizing the different summary measures using statistical methods. Second, articles from different meta-analyses may have overlapped and failed to include the original meta-analysis data. Third, this study included primarily a meta-analysis of the effect of hip protectors on the prevention of falls and hip fractures, leaving out the effect of hip protectors on fear of falling. It was found that fear of falling promoted adherence to hip protectors and increased self-efficacy for falls among institutionalized people [41, 42]. However, fewer studies have been conducted on community residents. Moreover, meta-analysis of the effects of hip protectors on fear of falling should be performed in the future. Fourth, the meta-analysis included in this study did not discuss the effects of soft hip protectors separately from those of hard hip protectors, which have been found to be different. Biomechanical comparison tests showed that only the hard hip protector was able to reduce the stress below the mean fracture threshold of 3100 N [43]. Future studies should consider comparing the efficacy of different hip protectors for fall prevention and hip fracture prevention. Fifth, the meta-analysis included in this study did not discuss the effect of the angle at the time of fall on hip fracture incidence. A study using biofidelic finite element models showed that the incidence of hip fractures was highest when the direction of impact was lateral, as was 15 degrees posterior [44]. And this angle was also in the greatest range of the hip protector attenuation in peak compressive stress [45]. In the future, researchers should consider incorporating a number of new technologies, including video-captured falls [46], which can discern the direction of the fall in aggregate. Sixth, the meta-analysis included in this study did not discuss the wearing position of the hip protector. An incorrect wearing position of the hip protector can also decrease the effectiveness of the protection. One study demonstrated that when worn in the correct position, the hip protector can attenuate stresses by 40%, but if the hip protector is moved 50 mm in the anterior, posterior, or lateral position (i.e., misalignment), the attenuation capacity is reduced to less than 20% [47].

## Conclusion

Wearing hip protectors is an effective way for preventing hip fractures in institutionalized older adults, but not in community-dwelling older adults.

### Electronic supplementary material

Below is the link to the electronic supplementary material.

Supplementary Material 1

Supplementary Material 2

## Data Availability

The datasets analysed during the current study are available from the corresponding author on reasonable request.
