# Supplementary material for: Does hip protector prevent falls and hip fractures? An umbrella review of meta-analyses
Source: BMC Geriatr. 2024 Jun 12;24:514. doi: 10.1186/s12877-024-05122-x (PMC11170778; doi:10.1186/s12877-024-05122-x)
Supplement: Supplementary file 1 — Supplementary Material 1 [file 12877_2024_5122_MOESM1_ESM.docx]

**References list**

**Included（N=6）：**

**Waldegger 2003**{published and unpublished data}

Waldegger L, Cranney A, Man-Son-Hing M, Coyle D. Cost-effectiveness of hip protectors in institutional dwelling elderly. OSTEOPOROSIS INTERNATIONAL. 2003;14(3): 243-250.DOI:10.1007/s00198-002-1354-3

**Sawka 2005**{published and unpublished data}

Sawka AM, Boulos P, Beattie K, et al. Do hip protectors decrease the risk of hip fracture in institutional and community-dwelling elderly? A systematic review and meta-analysis of randomized controlled trials. OSTEOPOROSIS INTERNATIONAL. 2005;16(12): 1461-1474.DOI:10.1007/s00198-005-1932-2

**Parker 2006**{published and unpublished data}

Parker MJ, Gillespie WJ, Gillespie LD. Effectiveness of hip protectors for preventing hip fractures in elderly people: systematic review. BMJ-British Medical Journal. 2006;332(7541): 571-573.DOI:10.1136/bmj.38753.375324.7C

**Sawka 2007**{published and unpublished data}

Sawka AM, Boulos P, Beattie K, et al. Hip protectors decrease hip fracture risk in elderly nursing home residents: a Bayesian meta-analysis. J Clin Epidemiol. 2007;60(4): 336-44.DOI:10.1016/j.jclinepi.2006.07.006

**Oliver D 2007**{published and unpublished data}

Oliver D, Connelly JB, Victor CR, et al. Strategies to prevent falls and fractures in hospitals and care homes and effect of cognitive impairment: systematic review and meta-analyses. Br Med J (Clin Res Ed). 2007; 334(7584): 82-85.DOI:10.1136/bmj.38753.375324.7C

**Santesso 2014**{published and unpublished data}

Santesso N, Carrasco‐Labra A, Brignardello‐Petersen R. Hip protectors for preventing hip fractures in older people. Cochrane Database of Systematic Reviews. 2014;(3):1 -81.DOI:10.1002/14651858.CD001255.pub5

**Excluded（N=27）**

Duplicates removed（N=7）

Excluded based on title and abstract N=7

**Pfeifer M 1999{**published and unpublished data}

Pfeifer M, Minne HW. Vitamin D and Hip Fracture. Trends in Endocrinology & Metabolism. 1999; 10(10): 417-420.DOI:10.1016/S1043-2760(99)00197-6

**Cranney A 2000**{published and unpublished data}

Cranney A, Welch V, Adachi J, et al. Calcitonin for preventing and treating corticosteroid‐induced osteoporosis. Cochrane Database of Systematic Reviews. 2000; (1): -.DOI:10.1002/14651858.CD001983

**Alguacil 2003**{published and unpublished data}

Alguacil IM, Máximo N. Protector de cadera: una prevención infravalorada. Revista Española de Geriatría y Gerontología. 2003; 38(5): 288-293.DOI:10.1016/S0211-139X(03)74900-2

**Jung 2009**{published and unpublished data}

Jung D, Lee J, Lee SM. A Meta-Analysis of Fear of Falling Treatment Programs for the Elderly. WESTERN JOURNAL OF NURSING RESEARCH. 2009; 31(1): 6-16.DOI:10.1177/0193945908320466

**Clark 2015**{published and unpublished data}

Clark L, Fairhurst C, Cook E, TorgersOn DJ. Important outcome predictors showed greater baseline heterogeneity than age in two systematic reviews. JOURNAL OF CLINICAL EPIDEMIOLOGY. 2015; 68(2): 175-181.DOI:10.1016/j.jclinepi.2014.09.023

**Stubbs 2015**{published and unpublished data}

Stubbs B, Denkinger MD, Brefka S, Dallmeier D. What works to prevent falls in older adults dwelling in long term care facilities and hospitals? An umbrella review of meta-analyses of randomised controlled trials. MATURITAS. 2015; 81(3): 335-342.DOI:10.1016/j.maturitas.2015.03.026

**Korall 2015**{published and unpublished data}

Korall AMB, Feldman F, Scott VJ, et al. Facilitators of and Barriers to Hip Protector Acceptance and Adherence in Long-term Care Facilities: A Systematic Review. Journal of the American Medical Directors Association. 2015; 16(3): 185-193.DOI:10.1016/j.jamda.2014.12.004

Without available full-text(N=1)

**Sawkar 2005{**published and unpublished data}

Sawka AM, Boulos P, Beattie K, et al. A meta-analysis of hip protector trials in institutionalized elderly using a Bayesian approach. JOURNAL OF BONE AND MINERAL RESEARCH. 2005; 20(9): S290-S290.DOI:

Excluded based on full-text N=12

N=10-no meta analysis

**Norton 1999** {published and unpublished data}

Norton R. Preventing falls and fall-related injuries among older people. AUSTRALASIAN JOURNAL ON AGEING. 1999. 18(4): 160-166.DOI:10.1111/j.1741-6612.1999.tb00121.x

**McClure 2005**{published and unpublished data}

McClure RJ, Turner C, Peel N, Spinks A, Eakin E, Hughes K. Population‐based interventions for the prevention of fall‐related injuries in older people. Cochrane Database of Systematic Reviews. 2005; (1): -.DOI:10.1002/14651858.CD004441.pub2

**Kannus 2005**{published and unpublished data}

Kannus P, Sievänen H, Palvanen M, Järvinen T, Parkkari J. Prevention of falls and consequent injuries in elderly people. The Lancet. 2005; 366(9500): 1885-1893.DOI:10.1016/S0140-6736(05)67604-0

**Close 2005**{published and unpublished data}

Close JCT, Lord SL, Menz HB, Sherrington C. What is the role of falls. OsteoporosisBest Practice & Research Clinical Rheumatology. 2005; 19(6): 913-935.DOI:10.1016/j.berh.2005.06.002

**Mak 2010**{published and unpublished data}

Mak J, Cameron ID, March LM. Evidence-based guidelines for the management of hip fractures in older persons: an update. MEDICAL JOURNAL OF AUSTRALIA. 2010; 192(1): 37-41.DOI:

**Oliver 2010**{published and unpublished data}

Oliver D, Healey F, Haines TP. Preventing Falls and Fall-Related Injuries in Hospitals. Falls and Their PreventionClinics in Geriatric Medicine. 2010; 26(4): 645-692.DOI:10.1016/j.cger.2010.06.005

**Neyens 2011**{published and unpublished data}

Neyens JC, van Haastregt JC, Dijcks BP, et al. Effectiveness and Implementation Aspects of Interventions for Preventing Falls in Elderly People in Long-Term Care Facilities: A Systematic Review of RCTs. Journal of the American Medical Directors Association. 2011; 12(6): 410-425.DOI:10.1016/j.jamda.2010.07.018

**de Bot 2015**{published and unpublished data}

de Bot R, Veldman HD, Witlox AM, van Rhijn LW, Hiligsmann M. Hip protectors are cost-effective in the prevention of hip fractures in patients with high fracture risk. OSTEOPOROSIS INTERNATIONAL. 2020; 31(7): 1217-1229.DOI:10.1016/j.maturitas.2015.03.026

**Montero-Odasso 2021**{published and unpublished data}

Montero-Odasso MM, Kamkar N, Pieruccini-Faria F, et al. Evaluation of Clinical Practice Guidelines on Fall Prevention and Management for Older Adults A Systematic Review. JAMA Network Open. 2021; 4(12): -.DOI:10.1001/jamanetworkopen.2021.38911

**Andre 2022**{published and unpublished data}

Andre J, Goes MM, Oliveira H, Vieira JV, Santos M. Surveillance and Fall Prevention in the Elderly. GERONTECHNOLOGY IV. 2022; 355-365.DOI:10.1007/978-3-030-97524-1_33

N=1-no systematic review

**Sawka 2007{**published and unpublished data}

Sawka AM, Gafni A, Boulos P, et al. Could a policy of provision of hip protectors to elderly nursing home residents result in cost savings in acute hip fracture care? The case of Ontario, Canada. OSTEOPOROSIS INTERNATIONAL. 2007; 18(6): 819-827.DOI:10.1007/s00198-006-0307-7

N=1-not relevant

**Lin 2006**{published and unpublished data}

Lin JT, Lane JM. Rehabilitation of the Older Adult with an Osteoporosis-Related Fracture. Geriatric RehabilitationClinics in Geriatric Medicine. 2006; (2): 435-447.DOI:10.1016/j.cger.2005; 12.010
